# Impact of clinically tested NEP/ACE inhibitors on tumor uptake of [^111^In-DOTA]MG11—first estimates for clinical translation

**DOI:** 10.1186/s13550-015-0158-3

**Published:** 2016-02-16

**Authors:** Aikaterini Kaloudi, Berthold A. Nock, Emmanouil Lymperis, Roelf Valkema, Eric P. Krenning, Marion de Jong, Theodosia Maina

**Affiliations:** Molecular Radiopharmacy, INRASTES, NCSR “Demokritos”, Ag. Paraskevi Attikis, GR-153 10 Athens, Greece; Department of Nuclear Medicine, Erasmus MC, 3015 GD Rotterdam, the Netherlands; Department of Radiology, Erasmus MC, 3015 GD Rotterdam, the Netherlands

**Keywords:** NEP inhibition, ACE inhibition, In situ radioligand stabilization, Tumor targeting, Enhancement of tumor uptake, [^111^In-DOTA]MG11

## Abstract

**Background:**

We have recently shown that treatment of mice with the neutral endopeptidase (NEP) inhibitor phosphoramidon (PA) improves the bioavailability and tumor uptake of biodegradable radiopeptides. For the truncated gastrin radiotracer [^111^In-DOTA]MG11 ([(DOTA)DGlu^10^]gastrin(10–17)), this method led to impressively high tumor-to-kidney ratios. Translation of this concept in the clinic requires the use of certified NEP inhibitors, such as thiorphan (TO) and its orally administered prodrug racecadotril (Race). Besides NEP, angiotensin-converting enzyme (ACE) has also been implicated in the catabolism of gastrin analogs. In the present study, we first compared the effects induced by NEP inhibition (using PA, TO, or Race) and/or by ACE inhibition (using lisinopril, Lis) on the biodistribution profile of [^111^In-DOTA]MG11 in mice. In addition, we compared the efficacy of PA and TO at different administered doses to enhance tumor uptake.

**Methods:**

[^111^In-DOTA]MG11 was coinjected with (a) vehicle, (b) PA (300 μg), (c) TO (150 μg), (d) Lis (100 μg), (e) PA (300 μg) plus Lis (100 μg), or (f) 30–40 min after intraperitoneal (ip) injection of Race (3 mg) in SCID mice bearing AR42J xenografts. In addition, [^111^In-DOTA]MG11 was coinjected with vehicle, or with progressively increasing amounts of PA (3, 30, or 300 μg) or TO (1.5, 15, and 150 μg) in SCID mice bearing twin A431-CCK2R(+/−) tumors. In all above cases, biodistribution was conducted at 4 h postinjection (pi).

**Results:**

During NEP inhibition, the uptake of [^111^In-DOTA]MG11 in the AR42J tumors impressively increased from 1.8 ± 1.0 % ID/g (controls) to 15.3 ± 4.7 % ID/g (PA) and 12.3 ± 3.6 % ID/g (TO), while with Race tumor values reached 6.8 ± 2.8 % ID/g. Conversely, Lis had no effect on tumor uptake and no additive effect when coinjected with PA. During the dose dependence study in mice, PA turned out to be more efficacious in enhancing tumor uptake of [^111^In-DOTA]MG11 in the CCK2R-positive tumors compared to equimolar amounts of TO. In all cases, renal accumulation remained low, resulting in notable increases of tumor-to-kidney ratios.

**Conclusions:**

This study has confirmed NEP as the predominant degrading enzyme of [^111^In-DOTA]MG11 and ruled out the involvement of ACE in the in vivo catabolism of the radiotracer. NEP inhibition with the clinically tested NEP inhibitors TO and Race resulted in significant enhancement of tumor-to-kidney ratios vs. controls. However, compared with PA, TO and its prodrug Race induced less potent increases of tumor uptake, highlighting the significance of inhibitor type, administration route, and dose for implementing a first proof-of-principle study in human.

**Electronic supplementary material:**

The online version of this article (doi:10.1186/s13550-015-0158-3) contains supplementary material, which is available to authorized users.

## Background

Cholecystokinin subtype 2 receptors (CCK2Rs) have attracted much attention in nuclear oncology due to their overexpression in various human tumors, including medullary thyroid carcinoma (MTC), small cell lung cancer, astrocytomas, and stromal ovarian cancers [1–4]. Accordingly, peptide radioligands based on gastrin or on cholecystokinin (CCK) have been proposed for diagnostic imaging and radionuclide therapy of CCK2R-positive tumors [5, 6].

**Fig. 1:**
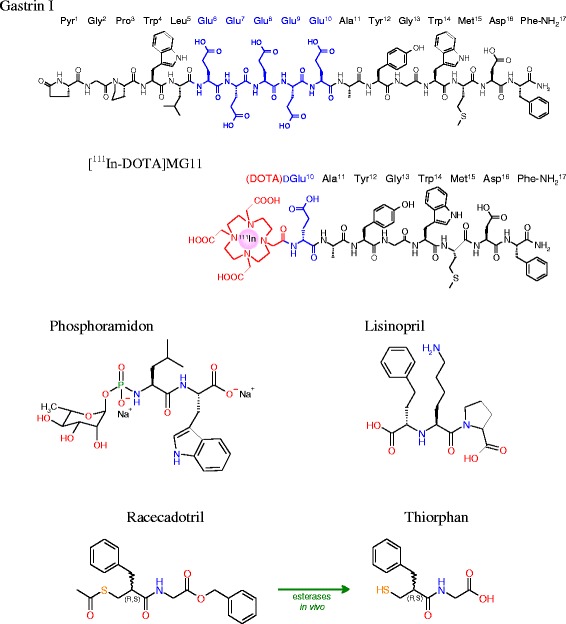
Human gastrin I; [^111^In-DOTA]MG11; the NEP inhibitors PA, TO, and its prodrug Race; and the ACE inhibitor Lis

Human gastrin I is a C-terminally amidated heptadecapeptide characterized by a penta-Glu repeat in positions 6 through 10 (pGlu-Gly-Pro-Trp-Leu-(Glu)_5_-Ala-Tyr-Gly-Trp-Met-Asp-Phe-NH_2_; Fig. 1) [7]. This highly polar and negatively charged amino acid sequence determines important biological properties of gastrin and its radiolabeled analogs. Thus, Glu^6–10^-containing radioligands show high receptor affinity and are metabolically robust. As a result, they efficiently localize in CCK2R-rich lesions in rodents and in patients, but their application in the clinic has been hitherto restricted by their unfavorably high renal retention. Conversely, truncated *des*Glu^6–10^ gastrin analogs, as for example [^111^In-DOTA]MG11 ([(DOTA)DGlu^10^]gastrin(10–17); Fig. 1), display favorably low renal uptake but also severely impaired ability to target CCK2R-positive tumors in vivo, owing to their poor metabolic stability [6, 8].

According to previous reports, proteases that might be implicated in the degradation of CCK and gastrin analogs include (a) aminopeptidases [9], (b) angiotensin-converting enzyme (ACE, EC 3.4.15.1) [10], and (c) neutral endopeptidase (NEP, neprilysin, EC 3.4.24.11) [11, 12]. Given that N-terminal capping is an effective means to prevent peptide degradation by aminopeptidases [9, 13], it is reasonable to assume that [^111^In-DOTA]MG11 with a bulky radiometal chelate attached at the N-terminus becomes aminopeptidase-resistant. The role of ACE in the degradation of CCK and different-length gastrin analogs has been previously investigated in vitro [10]. Interestingly, minigastrin (MG, gastrin(5–17)) was shown to be resistant to ACE, whereas successive shortening of the penta-Glu chain to truncated gastrin analogs with less than two Glu residues led to in vitro degradation by ACE. Accordingly, [^111^In-DOTA]MG11 is expected to be resistant to ACE by virtue of the polar ^111^In-DOTA^9^-DGlu^10^ sequence, mimicking the Glu^9–10^ construct of ACE-resistant gastrin(9–17). However, the role of ACE in the in vivo fate of gastrin analogs, including [^111^In-DOTA]MG11, has not been elucidated yet. The third protease implicated in the metabolism of gastrin is NEP, an ectoenzyme with broad substrate specificity and a wide distribution in the body. While scarcely found in plasma, NEP is abundantly expressed in most tissues, anchored on the endothelial cell surface of the vasculature compartment, and in major organs, such as kidneys, liver, lungs, and the gastrointestinal tract [14]. The involvement of NEP in the in vitro and in vivo degradation of gastrin has been well described in previous reports [11, 12].

We have recently proposed an effective strategy to improve the bioavailability and tumor uptake of biodegradable radiopeptides, involving in situ inhibition of the degrading protease(s) (e.g., NEP) by coinjection of a suitable inhibitor (e.g., the NEP inhibitor phosphoramidon, PA) [15–17]. For the truncated gastrin radiotracer [^111^In-DOTA]MG11, this method led to impressive in vivo stabilization, which translated into remarkably increased uptake in CCK2R-positive tumors in mice and into high tumor-to-kidney ratios [15, 18].

The above promising results have prompted further research toward clinical translation of the proposed concept in a first “proof-of-principle” study in man, using [^111^In-DOTA]MG11 as a paradigm. The success of this step largely relies on the identification of the peptidase(s) actually responsible for the proteolytic degradation of [^111^In-DOTA]MG11 after its entry in the circulation by intravenous injection. Rapid in vivo degradation deteriorates the radiotracer supply to target sites and eventually compromises its localization on tumor CCK2R-positive lesions. Therefore, we were first interested to study the potential involvement of ACE on the tumor uptake of [^111^In-DOTA]MG11. For this purpose, the effect of in situ ACE inhibition by lisinopril (Lis) [19] alone vs. controls was compared in mice bearing AR42J tumors. Furthermore, the combination of dual ACE/NEP inhibition after injection of a Lis and PA mixture vs. single NEP inhibition by PA was compared in the same mouse model.

Another important step toward clinical translation is the assessment of biosafety and efficacy of the NEP inhibitor intended for human application. It should be noted that so far, no extensive toxicity studies have been reported for PA. This bacterial origin and potent NEP inhibitor has been administered in human only in homeopathic amounts [16, 20]. Consequently, we decided to compare the in vivo efficacy of the clinically certified NEP inhibitor thiorphan (TO) [21, 22] vs. PA to enhance the tumor uptake of [^111^In-DOTA]MG11 in the same mouse model. Racecadotril (Race) is a prodrug of TO administered orally and a registered antidiarrheal drug (Fig. 1) [23, 24], which has also been included in our study. Race was administered intraperitoneally (ip) due to its poor water solubility prior to [^111^In-DOTA]MG11 iv injection.

In the last part of the present study, we have directly compared the efficacy of TO and PA iv-injected in equimolar and progressively increasing doses to enhance the uptake of [^111^In-DOTA]MG11 in a double A431-CCK2R(+/−) tumor mouse model.

## Methods

### Chemistry and radiochemistry

#### Chemicals and radionuclides

All chemicals were reagent grade and used without further purification. DOTA-MG11 (DOTA-minigastrin 11, DOTA-DGlu-Ala-Tyr-Gly-Trp-Met-Asp-Phe-NH_2_) and DG2 (Demogastrin 2, N_4_-Gly-DGlu-(Glu)_5_-Ala-Tyr-Gly-Trp-Met-Asp-Phe-NH_2_) [25] were purchased from PiChem (Graz, Austria). PA (phosphoramidon disodium dehydrate, *N*-(α-rhamnopyranosyloxyhydroxyphosphinyl)-*L*-leucyl-*L*-tryptophan × 2Na × 2H_2_O) was provided by PeptaNova GmbH (Sandhausen, Germany). TO (DL-thiorphan, DL-3-mercapto-2-benzylpropanoylglycine) was a kind gift of Prof. B. Roques (Université René Descartes, Paris, France). Lis (lisinopril dehydrate, ((*S*)1-1-[*N*2-(1-carboxy-3-phenylpropyl)-lysyl-proline dehydrate, MK 521) and Race (racecadotril, (*RS*)-benzyl *N*-[3-(acetylthio)-2-benzylpropanoyl]glycinate) were purchased from Sigma-Aldrich (Fig. 1).

Indium-111 used for labeling was purchased in the form of ^111^InCl_3_ in a solution of 0.05 M HCl (0.5 mL) from Mallinckrodt Medical B.V. (Petten, the Netherlands).

#### Preparation of [^111^In-DOTA]MG11

Lyophilized DOTA-MG11 was dissolved in water to a final concentration of 1 mM, and 50 μL aliquots were stored at −20 °C. Labeling with ^111^In was conducted in an Eppendorf vial containing 0.1 M sodium acetate buffer pH 4.6 in the presence of excess methionine (Met) to prevent oxidation of Met^15^ in DOTA-MG11 [26]. Freshly prepared sodium ascorbate buffer (10 mM) was added in the vial, followed by ^111^InCl_3_ solution (37–74 MBq), Met (1000 nmol), and DOTA-MG11 (10 nmol). The mixture was left to react at 90 °C for 20 min. Prior to performing quality control by HPLC, EDTA in 0.1 M acetate buffer (pH 4.6) was added to a final concentration of 1 mM to the labeling reaction mixture as a “free” ^111^In^3+^ scavenger.

#### Quality control of [^111^In-DOTA]MG11

For quality control of the labeled reaction mixture, RP-HPLC was performed using system 1: A Waters Chromatograph (Waters, Vienna, Austria) based on a 600 solvent delivery system coupled to a Waters 996 photodiode array UV detector and a Gabi gamma detector (Raytest, RSM Analytische Instrumente GmbH, Germany) employing a 20 μL injection loop was applied. The Millennium Software (Waters, USA) was used for data processing and chromatographic control, and an XTerra RP-18 (5 μm, 4.6 mm × 150 mm) cartridge column (Waters, Germany) was eluted at 1 mL/min flow rate with a linear gradient starting from 0 % B and advancing to 40 % B within 40 min (solvent A = 0.1 % aqueous trifluoroacetic acid (TFA) and B = MeCN). For metabolism studies, HPLC analysis was performed using system 2: A Waters Chromatograph (Waters, Vienna, Austria) with a 600E multisolvent delivery system coupled to a Gabi gamma detector (Raytest, Germany) employing a 0.5-mL injection loop was applied. Data processing and chromatography run were controlled with Empower Software, and a Symmetry Shield RP-18 (5 μm, 3.9 mm × 20 mm) column (Waters, Germany) was eluted adopting linear gradient starting from 0 % B and advancing to 40 % B within 40 min (solvent A = 0.1 % aqueous TFA and B = MeCN) with a flow rate of 1 mL/min. Radioactivity measurements were conducted in an automated well-type gamma counter (NaI(Tl) crystal, Canberra Packard Auto-Gamma 5000 series instrument) calibrated for ^111^In.

### Biology

#### Cell lines and cell culture

The rat pancreatic tumor cell line AR42J, endogenously expressing the CCK2R [27], was kindly provided by Prof. C. Decristoforo (University Clinic Innsbruck, Austria). The human epidermoid carcinoma A431 cell line transfected to stably express the CCK2R (A431-CCK2R(+)) or devoid of CCK2R expression (A431-CCK2R(−)) was a gift from Prof. O. Boerman (Department of Nuclear Medicine, Radboud University Nijmegen Medical Centre, Nijmegen, The Netherlands) and Prof. L. Aloj (Istituto di Biostrutture e Bioimmagini, Consiglio Nazionale delle Ricerche, Naples, Italy) [28].

All culture media were purchased from Gibco BRL, Life Technologies (Grand Island, NY, USA), and supplements were supplied by Biochrom KG Seromed (Berlin, Germany). AR42J cells were cultured in F-12K Nutrient Mixture (Kaighn’s Modification), supplemented with 10 % (*v*/*v*) fetal bovine serum, 100 U/mL penicillin, 100 μg/mL streptomycin, and 1 mM L glutamine. A431-CCK2R(+/−) cells were grown in Dulbecco’s Modified Eagle medium with GlutaMAX-I supplemented with 10 % (*v*/*v*) fetal bovine serum, 100 U/mL penicillin, 100 μg/mL streptomycin, 4500 mg/L D-glucose, and 250 μg/mL G418. Cells were kept in a controlled humidified air containing 5 % CO_2_ at 37 °C. Splitting of cells with a ratio of 1:2 to 1:5 was performed when approaching confluency using a trypsin/EDTA solution (0.05/0.02 % *w*/*v*) [29].

#### Metabolism in blood

Animal experiments were carried out in compliance with European and national regulations and were approved by national authorities. For metabolic studies, in-house male Swiss albino mice (30 ± 5 g) were used. A bolus containing [^111^In-DOTA]MG11 (100 μL, 11–22 MBq, 3 nmol of total peptide, normal saline/EtOH 9/1 *v*/*v*) was injected in the tail vein of mice, together with (a) vehicle (100 μL; control), (b) PA (100 μL of vehicle containing 300 μg PA; PA), or (c) (100 μL of vehicle containing 150 μg TO; TO). Additional animals intraperitoneally (ip) received a fine dispersed suspension of Race (3 mg Race dissolved in 0.025 mL DMSO and freshly mixed with 0.375 mL saline) 30–40 min prior to radioligand injection. The animals were kept for 5 min in cages with free access to water. They were sacrificed by cardiac puncture under ether anesthesia, and blood was withdrawn with a syringe and immediately placed in a pre-chilled polypropylene vial on ice containing EDTA and Met. Blood samples were centrifuged at 2000*g* at 4 °C for 10 min. The plasma was collected, an equal volume of MeCN was added, and the mixture was centrifuged for 10 min at 15,000*g* at 4 °C. The supernatant was collected and concentrated under a gentle N_2_-flux at 40 °C to a volume of ≈0.1 mL; the concentrate was diluted with physiological saline (0.4 mL) and passed through a Millex-GV syringe-driven filter unit (0.22 μm/13 mm; Millipore, Milford, USA). Suitable aliquots of the filtrate were analyzed by RP-HPLC [15, 29]. The *t*_R_ of the parent radiopeptide in the applied chromatographic conditions (system 2) was established by coinjection of samples with [^111^In-DOTA]MG11.

#### Biodistribution in AR42J tumor-bearing SCID mice

In-house male SCID mice (NCSR “Demokritos” Animal House) of 6 weeks of age at the time of arrival (18 ± 2 g body weight) were inoculated subcutaneously (sc) in their flanks with a suspension of freshly harvested AR42J cells (1 × 10^7^ cells in ~150 μL saline). Animals were kept in aseptic conditions for 14 days when well-palpable tumors developed at the inoculation sites (0.31 ± 0.17 g) [29]. At the day of biodistribution, animals received a bolus of [^111^In-DOTA]MG11 (100 μL, 37–74 kBq, 10 pmol total peptide, in saline/EtOH 9/1 *v*/*v*) through the tail vein, coinjected with (a) vehicle (100 μL; control group, *n* = 10), (b) PA (100 μL of vehicle containing 300 μg PA; PA group, *n* = 10), (c) TO (100 μL of vehicle containing 150 μg TO; TO group, *n* = 4), (d) Lis (100 μL of vehicle containing 100 μg Lis; Lis group, *n* = 4), (e) PA plus Lis (100 μL vehicle containing 300 μg PA and 100 μg Lis; PA+Lis group, *n* = 5), or (f) 30–40 min after ip injection of Race (3 mg Race dissolved in 0.025 mL DMSO and freshly mixed with 0.375 mL saline; Race group, *n* = 4). In a separate animal group, the mice were coinjected with 100 μL vehicle containing both 300 μg PA and 100 μg DG2 [25] to assess non-specific tumor uptake during in situ NEP inhibition (in vivo CCK2R blockade; block group, *n* = 4).

Mice had access to drinking water ad libitum until they were euthanized at 4 h pi. Blood samples, organs of interest, and tumors were collected immediately after dissection, weighted, and measured for radioactivity in the gamma counter; only stomachs were emptied of their contents prior to measurements. Biodistribution data were calculated as percent of injected dose per gram tissue (% ID/g) with the aid of suitable standards of the injected dose, using the GraphPad Prism Software (San Diego, CA).

#### PA and TO dose dependence study in SCID mice bearing twin A431-CCK2R(+/−) tumors

Inocula of freshly harvested A431-CCK2R(+/−) cells (1.6 × 10^7^/1.4 × 10^7^ cell suspensions in 150 μL saline) were sc-injected in the right and left flanks of the SCID mice (male SCID mice, 6 weeks of age and of 18 ± 2 g body weight on arrival day; NCSR “Demokritos” Animal House). The animals were kept in aseptic conditions for 8 days till well-palpable tumors (0.26 ± 0.08 g) developed at the inoculation sites [29]. At the day of the experiment, the mice received a bolus of [^111^In-DOTA]MG11 (100 μL, 37–74 kBq, 10 pmol total peptide, in saline/EtOH 9/1 *v*/*v*) through the tail vein, coinjected with (a) vehicle (100 μL; control group, *n* = 5), (b) three different doses of PA (3 μg, *n* = 5; 30 μg, *n* = 4; or 300 μg PA, *n* = 10, dissolved in 100 μL vehicle), and (c) three different doses of TO (1.5, 15, or 150 μg TO dissolved in 100 μL vehicle, all groups of *n* = 4). Mice had free access to drinking water until they were sacrificed at 4 h pi, and biodistribution was conducted as described above.

#### Statistical analysis

The in vivo data were statistically analyzed with the Student’s *t* test (Prism™ 2.01, GraphPad Software, San Diego, CA). Analyses were two-tailed and a *P* value <0.05 was considered statistically significant.

## Results

### Radiolabeling

The radiolabeling of DOTA-MG11 with ^111^In was straightforward following published methods and involved 20-min incubation of the peptide conjugate in acidic medium at 90 °C in the presence of ^111^InCl_3_ [26]. A >96 % radiometal incorporation was typically demonstrated by RP-HPLC analysis (system 1). The radiochemical purity was >97 %, verifying that the addition of Met in the labeling reaction mixture prevented the oxidation of Met^15^ in the peptide chain (Additional file 1: Figure S1).

#### Metabolism in blood

**Fig. 2:**
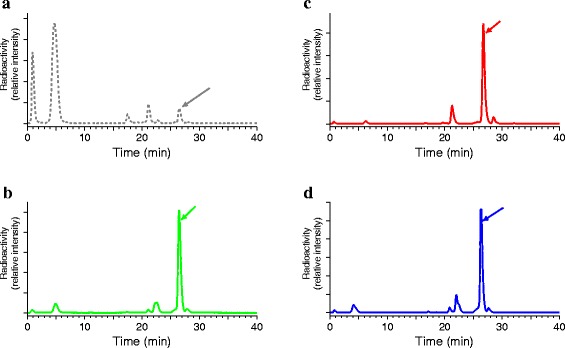
Stability of [^111^In-DOTA]MG11 in peripheral mouse blood at 5 min pi. Analysis by HPLC (system 2) of murine blood collected 5 min after injection of [^111^In-DOTA] MG11 with **a** vehicle (<4 % intact radiotracer), **b** PA (300 μg; >70 % intact radiopeptide), **c** TO (150 μg; >70 % intact radiopeptide), or **d** 30–40 min after ip injection of Race (2 mg; >70 % intact radiopeptide). The *t*_R_ of [^111^In-DOTA]MG11 is indicated by the *arrow*

As shown by HPLC analysis of mouse blood collected 5 min pi, [^111^In-DOTA]MG11 was very rapidly degraded in vivo in agreement with previous findings (Fig. 2a) [8, 15, 26]. Treatment of mice with PA, TO, and its ip pre-administered prodrug Race notably increased the amount of intact radiotracer in peripheral blood from <5 to >70 % (Fig. 2b, c, d, respectively). Conversely, coinjection of [^111^In-DOTA] MG11 with the ACE inhibitor Lis produced no change on radiotracer stability in mouse circulation up to 5 min pi (results not shown), in support of the assumption that [^111^In-DOTA]MG11 is resistant to ACE [18].

#### Biodistribution in AR42J tumor-bearing mice—impact of NEP/ACE inhibitors

**Fig. 3:**
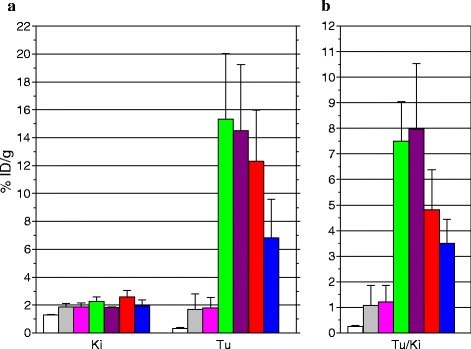
**a** Uptake of [^111^In-DOTA]MG11 in kidneys and AR42J tumors at 4 h pi in SCID mice. Data are given for kidneys and tumors as mean of %ID/g ± SD, n ≥ 4; [^111^In-DOTA]MG11 iv-coinjection with vehicle (), with 100 μg Lis (), with 300 μg PA (), with a mixture of 100 μg Lis and 300 μg PA (), with 150 μg TO (), or 30–40 min after ip injection of 3 mg Race (); for CCK2R blockade, 40 nmol DG2 [25] was iv-coinjected along with 300 μg PA (). **b** Corresponding tumor-to-kidney ratios (Tu/Ki)

Results of [^111^In-DOTA]MG11 biodistribution in SCID mice bearing AR42J tumors at 4 h pi are summarized in Table 1 and in Fig. 3, as % ID/g (n ≥ 4). In addition to the values obtained by coinjection of the radiotracer with vehicle (controls), Table 1 includes values obtained during treatment of mice with one of the NEP inhibitors (PA, TO, and Race) and/or the ACE inhibitor Lis. In all cases, the radiotracer rapidly cleared from background tissues via the kidneys and the urinary tract with consistently low renal values. Uptake in the AR42J tumors remarkably increased from 1.82 ± 1.25 %ID/g in controls to 15.32 ± 4.71 % ID/g in the PA group (*P* < 0.001). Most importantly, this increase was shown to be receptor specific, as suggested by the significantly reduced tumor values determined during coinjection of excess DG2 [25] and PA (0.34 ± 0.04 % ID/g, *P* < 0.001). In contrast, coinjection of the ACE inhibitor Lis did not provoke any raise in tumor values (1.80 ± 0.74 % ID/g) vs. controls (*P* > 0.05), whereas coinjection of both peptidase inhibitors PA and Lis did not provoke any additional increase in the PA group values (14.51 ± 4.73 % ID/g, *P* > 0.05). This finding is a strong indication that ACE is not involved in the in vivo catabolism of [^111^In-DOTA]MG11.

**Table 1:** Cumulative biodistribution data of [^111^In-DOTA]MG11 in AR42J tumor-bearing SCID mice including controls and animals treated with NEP and/or ACE inhibitors

Organs	[^111^In-DOTA]MG11 (%ID/g tissue ± SD at 4 h pi, *n* ≥ 4)						
	+DG2 + PA^a^	Control	+Lis^b^	+PA^c^	+PA + Lis^d^	+TO^e^	+Race^f^
Blood	0.02 ± 0.01	0.04 ± 0.01	0.05 ± 0.02	0.06 ± 0.04	0.05 ± 0.02	0.02 ± 0.01	0.20 ± 0.08
Liver	0.14 ± 0.02	0.13 ± 0.03	0.13 ± 0.01	0.15 ± 0.04	0.17 ± 0.09	0.17 ± 0.05	1.19 ± 0.03
Heart	0.02 ± 0.01	0.05 ± 0.02	0.05 ± 0.01	0.07 ± 0.03	0.07 ± 0.03	0.03 ± 0.02	0.13 ± 0.03
Kidneys	1.31 ± 0.03	1.86 ± 0.25	1.85 ± 0.30	2.26 ± 0.33	1.82 ± 0.06	2.95 ± 0.80	1.93 ± 0.46
Stomach	0.08 ± 0.01	1.53 ± 0.65	2.83 ± 0.68	5.13 ± 2.29	6.21 ± 2.73	2.90 ± 0.51	4.60 ± 1.09
Intestines	0.48 ± 0.08	0.89 ± 0.76	0.65 ± 0.25	0.69 ± 0.13	0.82 ± 0.26	2.16 ± 0.88	0.83 ± 0.12
Spleen	0.09 ± 0.02	0.17 ± 0.07	0.34 ± 0.30	0.20 ± 0.08	0.30 ± 0.13	0.14 ± 0.04	0.30 ± 0.06
Muscle	0.01 ± 0.01	0.04 ± 0.01	0.04 ± 0.01	0.09 ± 0.17	0.07 ± 0.04	0.02 ± 0.01	0.10 ± 0.03
Lung	0.05 ± 0.01	0.07 ± 0.02	0.07 ± 0.01	0.10 ± 0.01	0.28 ± 0.19	0.12 ± 0.05	0.12 ± 0.03
Femur	0.04 ± 0.01	0.09 ± 0.05	0.08 ± 0.01	0.12 ± 0.06	0.15 ± 0.04	0.05 ± 0.01	0.26 ± 0.22
Pancreas	0.08 ± 0.01	0.11 ± 0.03	0.18 ± 0.09	0.36 ± 0.17	0.53 ± 0.24	0.30 ± 0.16	0.30 ± 0.061
Tumor	0.34 ± 0.04	1.82 ± 1.25	1.80 ± 0.74	15.32 ± 4.71	14.51 ± 4.73	12.32 ± 3.66	6.81 ± 2.79

^a^Coinjection of 50 nmol DG2 [25] and 300 μg PA to assess non-specific tumor uptake

^b^Coinjection of 100 μg Lis

^c^Coinjection with 300 μg PA

^d^Coinjection with 300 μg PA and 100 μg Lis

^e^Coinjection with 150 μg TO

^f^Radiotracer injection 30–40 min after ip injection of 3 mg Race

On the other hand, coinjection of TO, at a dose equimolar to PA, produced similar albeit slightly less potent increase of AR42J tumor uptake (12.32 ± 3.66 % ID/g, *P* > 0.05). Interestingly, the TO prodrug Race although administered at a ten times higher dose than TO induced half as high an increase in the tumor uptake of [^111^In-DOTA]MG11 (6.81 ± 2.79 %ID/g, *P* < 0.001).

#### Biodistribution in A431-CCK2R(+/−) tumor-bearing mice—impact of PA and TO dose

**Fig. 4:**
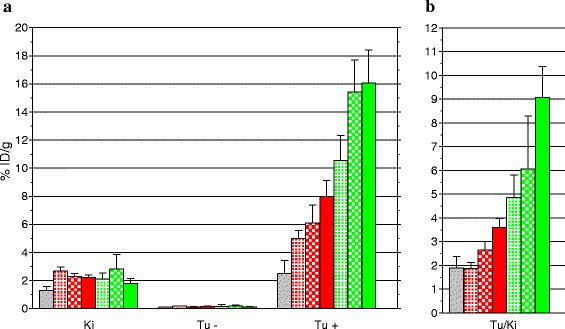
**a** Uptake of [^111^In-DOTA]MG11 in kidneys and A431-CCK2R(+) and A431-CCK2R(−) tumors at 4 h pi in SCID mice for controls and increasing amounts of the NEP inhibitors TO and PA. Data are given as mean of %ID/g ± SD, *n* ≥ 4. [^111^In-DOTA]MG11 iv-coinjection with vehicle (), with 1.5 μg TO (), with 15 μg TO (), with 150 μg TO (), with 3 μg PA (), with 30 μg PA (), or 300 μg PA () in SCID mice bearing twin A431-CCK2R(+) (Tu+) and naïve A431-CCK2R(−) (Tu−) human xenografts. **b** Corresponding tumor-to-kidney ratios (Tu/Ki)

The efficacy of the two NEP inhibitors PA and TO in improving the in vivo targeting of [^111^In-DOTA]MG11 was compared at 4 h pi in SCID mice bearing double A431-CCK2R(+/−) tumors. Three gradually increasing inhibitor doses and equimolar for PA (3, 30, and 300 μg) and TO (1.5, 15, and 150 μg) were tested, and the respective results are summarized in Table 2 and Fig. 4. It is interesting to note that in all cases, a significant and receptor-specific increase was recorded in the uptake of the radiotracer in the CCK2R-expressing tumors (*P* < 0.001), whereas no change was observed in the tumors devoid of CCK2R expression. Stepwise increase of TO dose resulted in significant and gradual increase of tumor uptake vs. controls. In the case of PA, however, increasing the dose from 30 to 300 μg did not further improve tumor uptake. Overall, PA clearly exerted a more potent tumor enhancement effect compared to the respective equimolar doses of TO (*P* < 0.001).

**Table 2:** Comparative biodistribution data of [^111^In-DOTA]MG11 in SCID mice bearing double A431-CCK2R(+/−) tumors at 4 h pi, including controls and animals coinjected with gradually increasing amounts of TO and PA; TO and PA injected doses were equimolar

Organs	[^111^In-DOTA]MG11 (%ID/g tissue ± SD at 4 h pi, *n* ≥ 4)						
	Control	+TO			+PA		
		1.5 μg	15 μg	150 μg	3 μg	30 μg	300 μg
Blood	0.01 ± 0.01	0.02 ± 0.01	0.03 ± 0.02	0.02 ± 0.01	0.02 ± 0.01	0.04 ± 0.01	0.02 ± 0.01
Liver	0.11 ± 0.02	0.16 ± 0.02	0.15 ± 0.02	0.16 ± 0.01	0.13 ± 0.03	0.13 ± 0.02	0.12 ± 0.01
Heart	0.02 ± 0.01	0.03 ± 0.00	0.03 ± 0.00	0.03 ± 0.01	0.02 ± 0.01	0.04 ± 0.01	0.03 ± 0.01
Kidneys	1.30 ± 0.29	2.67 ± 0.30	2.28 ± 0.21	2.22 ± 0.19	2.11 ± 0.45	2.81 ± 1.03	1.78 ± 0.37
Stomach	1.32 ± 0.26	2.59 ± 0.40	3.38 ± 0.20	4.01 ± 0.20	2.68 ± 0.67	3.19 ± 0.12	4.56 ± 1.16
Intestines	0.83 ± 0.53	0.43 ± 0.10	0.47 ± 0.04	0.48 ± 0.02	0.40 ± 0.17	0.32 ± 0.09	0.47 ± 0.20
Spleen	0.06 ± 0.01	0.12 ± 0.02	0.10 ± 0.01	0.14 ± 0.04	0.09 ± 0.04	0.10 ± 0.02	0.07 ± 0.01
Muscle	0.02 ± 0.01	0.03 ± 0.01	0.02 ± 0.01	0.03 ± 0.02	0.03 ± 0.01	0.04 ± 0.01	0.02 ± 0.01
Lung	0.03 ± 0.01	0.05 ± 0.00	0.05 ± 0.00	0.62 ± 0.02	0.07 ± 0.05	0.07 ± 0.01	0.07 ± 0.03
Femur	0.02 ± 0.01	0.05 ± 0.00	0.06 ± 0.00	0.09 ± 0.07	0.04 ± 0.03	0.05 ± 0.01	0.06 ± 0.01
Pancreas	0.05 ± 0.02	0.14 ± 0.03	0.12 ± 0.03	0.22 ± 0.04	0.14 ± 0.07	0.17 ± 0.02	0.20 ± 0.03
Tumor (−)	0.12 ± 0.02	0.18 ± 0.03	0.13 ± 0.01	0.17 ± 0.04	0.17 ± 0.0.13	0.18 ± 0.07	0.13 ± 0.02
Tumor (+)	2.50 ± 0.92	4.98 ± 0.58	6.10 ± 1.26	7.99 ± 1.12	10.55 ± 1.77	15.41 ± 2.28	16.05 ± 2.37

## Discussion

The advent of radiolabeled somatostatin analogs, like OctreoScan^®^ ([^111^In-DTPA]octreotide) and Lutathera^®^ ([^177^Lu-DOTA]Tate), in the management of sst_2_-expressing neuroendocrine tumors [30], has established the application of peptide radioligands for molecular imaging and radionuclide therapy of tumors. This success has largely depended on the metabolic robustness of the cyclic-octapeptide analogs applied. The evolution of peptide receptor-targeted diagnosis and therapy beyond the boundaries of sst_2_-positive neuroendocrine tumors is valid based on the overexpression of other peptide receptors in a variety of human tumors [31]. However, progress in this direction has been greatly restricted by the sub-optimal metabolic stability of peptide radioligands. Structural interventions to improve metabolic stability often deteriorate other important biological features of radiopeptides, as for example receptor affinity or pharmacokinetics [9].

We have recently proposed the in situ stabilization of biodegradable radiopeptides by coinjection of a suitable protease inhibitor, leading to significant improvement of tumor uptake [15]. This concept has indeed led to impressive diagnostic signal amplification on tumor lesions in preclinical models and was recently shown to improve the outcome of radionuclide therapy as well [32]. Especially in the case of [^111^In-DOTA]MG11 and other radiolabeled gastrins, high enhancement of tumor uptake combined with a preserved low kidney retention were observed, resulting in particularly appealing tumor-to-kidney ratios [18, 29]. These preclinical results, obtained after coinjection of [^111^In-DOTA]MG11 with the NEP inhibitor PA, warrant further validation in a “proof-of-principle” study in patients.

It should be noted, that our studies on in situ radiopeptide stabilization and enhancement of tumor uptake have primarily been focused on NEP inhibition by PA. Remarkably, a great variety of radioligands originating from different peptide families, including somatostatin, bombesin, and gastrin, have profited by application of this simple methodology, revealing a central role of NEP in initiating the in vivo degradation of these analogs [15]. The impact of in situ NEP inhibition relies on the abundant and ubiquitous presence of this ectoenzyme in the body combined with its broad substrate specificity [14]. However, NEP may not always exclusively determine the in vivo fate of radiopeptides. For example, ACE has often been implicated in the degradation of peptide ligands, and its role in the in vitro degradation of different-length gastrin analogs has been previously reported [10]. It should be stressed that the impact of in situ ACE inhibition on the bioavailability and tumor localization of [^111^In-DOTA]MG11 in vivo has not been thus far investigated.

For this purpose, in our study we have coinjected [^111^In-DOTA]MG11 together with the potent ACE inhibitor Lis, a registered antihypertensive drug [19]. As a result, we were not able to observe any change in the radioligand uptake in AR42J tumors in mice treated with Lis vs. controls at 4 h pi. In addition, dual NEP/ACE inhibition by combined coinjection of both Lis and PA inhibitors with [^111^In-DOTA]MG11 induced no additive effect vs. single NEP inhibition by PA (Table 1, Fig. 3; *P* > 0.05). These findings strongly suggest that ACE does not contribute to the in vivo processing of [^111^In-DOTA]MG11 and verify NEP as the major degrading protease.

Owing to the fast kinetics of radiopeptide localization to target sites upon entry in the bloodstream, only rapidly occurring degradation events—as those related to NEP—are relevant for tumor-targeting efficacy. Hence, the onset of NEP inhibition should occur and reach its maximum quite rapidly but not last longer than the time needed for the radioactivity to clear from blood and access the target. In this respect, the inhibitor should preferably induce rapid, complete, and transient NEP inhibition. In addition, it should be potent, water-soluble, chemically stable, and available at a reasonable cost. PA combines most of the above desirable properties; however, it has not been thoroughly investigated in terms of biosafety [17]. Only very low amounts of PA have been administered in human, not sufficient for in vivo effective inhibition of NEP [20].

Aiming at clinical translation, our interest has been attracted by two clinically certified NEP inhibitors, TO and its prodrug Race. TO is a potent, reversible NEP inhibitor with a relative rapid onset of action [21], but its water solubility is not ideal for iv injection as a bolus together with the radiopeptide. It has been previously tested in high doses in human, as for example by iv infusion of 150 mg in 250 mL of isotonic glucose in 42 patients showing excellent hemodynamic tolerance [22]. The impact of TO on the bioavailability and tumor uptake of our paradigm compound [^111^In-DOTA]MG11 was compared vs. PA 4 h after coinjection of the radiotracer with equimolar amounts of either inhibitor in AR42J tumor-bearing mice. As a result, we observed comparable and impressive enhancement of tumor values (Table 1, Fig. 3). On the other hand, Race is a registered antidiarrheal drug for oral use and a prodrug for TO [23, 24]. Due to its poor water solubility, it cannot be iv-administered in mice and was instead ip-injected in high molar excess (10-fold than the TO dose) 30–40 min prior to the iv injection of [^111^In-DOTA]MG11. As a result, tumor values significantly increased compared to controls but half as efficiently compared to the iv-injected hydrophilic inhibitors PA and TO. This finding highlights the significance of administration route and actual concentration of the inhibitor in the blood at the time of the radiotracer injection to achieve maximum enhancement of uptake on tumor lesions.

In the last part of this study, the efficacy of TO to enhance the uptake of [^111^In-DOTA]MG11 specifically in human CCK2R(+)-expressing tumors was compared vs. PA at progressively increasing administered doses in mice bearing double A431-CCK2R(+/−) tumors (Table 2, Fig. 4). While both NEP inhibitors induced significant and CCK2R-specific tumor uptake enhancement even at the lowest administered doses (1.5 μg TO and 3 μg PA), PA showed consistently superior efficacy at all tested dose levels compared to TO (*P* < 0.001). Remarkably, PA reached maximum tumor uptake enhancement at the 30 μg dose already, whereas TO reached half as high tumor values at the corresponding 10-fold molar dose of 150 μg. These intriguing findings may be tentatively assigned to the free thiol functionality of TO (Fig. 1). This strong nucleophile is prone to interact in vivo with electrophiles, and hence, the effective dose of TO might be much lower than originally anticipated. In fact, previous reports have demonstrated the fast in vivo inactivation of thiol-based NEP inhibitors, including TO [33, 34]. Another practical disadvantage of thiols is their propensity to oxidize in aqueous solutions to the respective disulfides, and hence, their storage for longer time periods in ready to use formulations for clinical application becomes challenging.

## Conclusions

In summary, translation of the concept of in situ stabilization of biodegradable peptide radioligands to improve tumor targeting and hence diagnostic sensitivity and therapeutic efficacy, from the preclinical setting into patients, poses certain challenges that need to be competently addressed. Promising data thus far obtained from mouse models for our paradigm radiotracer [^111^In-DOTA]MG11 and the NEP inhibitor PA have been directly compared herein with results retrieved after treatment of mice with the ACE inhibitor Lis and the NEP inhibitors TO and Race, all of which have been clinically tested previously. This study has shown that only NEP inhibition is relevant for clinical translation. It has also shown that significant enhancement of the radiotracer tumor uptake is indeed feasible by both TO and Race. However, for maximum efficacy, matching that of PA, further studies need to be performed to optimize dose, administration route, and time of inhibitor injection in respect to radiotracer injection and are currently actively pursued.

## Additional file

Additional file 1: Figure S1. Quality control of [^111^In-DOTA]MG11 labeling reaction product by HPLC. (DOCX 219 kb)

## Abbreviations

DOTA: 1,4,7,10-tetraazacyclododecane-1,4,7,10-tetraacetic acid
DTPA: diethylenetriamine-pentaacetic acid
N_4_: 6-(carboxyl)-1,4,8,11-tetraazaundecane
Octreotide: H_2_N-DPhe-c[Cys-Phe-DTrp-Lys-Thr-Cys]-Thr(ol)
PET: positron emission tomography
SPECT: single photon emission computed tomography
sst_2_: somatostatin subtype 2 receptor
Tate: H_2_N-DPhe-c[Cys-Tyr-DTrp-Lys-Thr-Cys]-Thr-OH
