# Supplementary material for: Impact of clinically tested NEP/ACE inhibitors on tumor uptake of [111In-DOTA]MG11—first estimates for clinical translation
Source: EJNMMI Res. 2016 Feb 16;6:15. doi: 10.1186/s13550-015-0158-3 (PMC4755954; doi:10.1186/s13550-015-0158-3)
Supplement: Additional file 1: Figure S1. — Quality control of [111In-DOTA]MG11 labeling reaction product by HPLC. (DOCX 219 kb) [file 13550_2015_158_MOESM1_ESM.docx]

**SUPPLEMENTARY FILE**

**Impact of clinically tested NEP/ACE inhibitors on tumor uptake of [^111^In-DOTA]MG11 – First estimates for clinical translation**

Aikaterini Kaloudi^1^, Berthold A. Nock^1^, Emmanouil Lymperis^1^, Roelf Valkema^2^, Eric P. Krenning^2^, Marion de Jong^2,3^, and Theodosia Maina^1^ ^🖃^

^1^Molecular Radiopharmacy, INRASTES, NCSR “Demokritos”, Ag. Paraskevi Attikis, GR-153 10 Athens, Greece; ^2^Department of Nuclear Medicine and ^3^Radiology, Erasmus MC, 3015 GD Rotterdam, The Netherlands

**Figure S1**. **Quality control of [^111^In-DOTA]MG11 labeling reaction product by HPLC.** Typical radiochromatogram of HPLC analysis (system 1) of [^111^In-DOTA]MG11 labeling reaction mixture, with *t*_R_ [^111^In-DOTA]MG11: 32 min (97.75% of total); *t*_R_ [^111^In-DOTA,MetO^15^]MG11: 26.5 min (0.87% of total); *t*_R_ of [^111^In]EDTA: 0.3 min (1.38% of total).
